# Amateurs exhibit greater psychomotor efficiency than novices: evidence from EEG during a visuomotor task

**DOI:** 10.3389/fpsyg.2025.1436549

**Published:** 2025-07-23

**Authors:** Guo Lu, John Elvis Hagan, Ming-Yang Cheng, Dong-Tai Chen, Chieh-Ju Lu, Fang-Yi Lin, Li-Ju Chen, Dan Li, Kuo-Pin Wang

**Affiliations:** ^1^International Football Education School, Jilin Agricultural University, Changchun, Jilin Province, China; ^2^Center for Cognitive Interaction Technology (CITEC), Bielefeld University, Bielefeld, Germany; ^3^Neurocognition and Action - Biomechanics Research Group, Faculty of Psychology and Sports Science, Bielefeld University, Bielefeld, Germany; ^4^Department of Sport Science Research, Taiwan Institute of Sports Science, Kaohsiung, Taiwan; ^5^Master Program of Sport Facility and Health Promotion, National Taiwan University, Taipei, Taiwan; ^6^Humanity and Sociology in Physical Education Department, Jilin Sport University, Changchun, China; ^7^NTU Plus Academy, National Taiwan University, Taipei, Taiwan

**Keywords:** neuromotor processes, motor performance, motor learning, attention, neurofeedback, brain oscillations, sensorimotor rhythm (SMR), mu rhythm

## Abstract

**Introduction:**

The goal of this study was to examine the neural activities, which contribute to performance efficiency in the early stages of motor skill learning, such as amateur versus novice. To achieve this goal, electroencephalography (EEG) was employed to compare the differences in EEG power that can be used to assess neural excitability between amateur and novice golfers during a visuomotor task (i.e., golf putting task).

**Methods:**

16 amateurs (9 females, 7 males, mean age = 20.81 ± 1.83; an intermediate skill level with an average handicap of 33 ± 5.68 and 3.81 ± 1.83 years of experience) and 16 novice golfers (9 females, 7 males, mean age = 22.25 ± 1.61; no prior experience in golf or formal training) were asked to perform a golf putting task while their EEG was recorded. During the warm-up session, each participant determined their individual putting distance, targeting a 40–60% success rate. Once established, participants were asked to perform 10 putts per block across 6 blocks in the experimental session.

**Results:**

The results of the study indicated that amateur golfers demonstrated: (1) higher Fz Theta power, (2) higher Fz, Pz, T7, T8 Alpha 2 power, (3) higher Mu 2 power, and (4) higher SMR power compared with novices during motor preparation. These findings suggest that amateur golfers exhibited reduced motor programming (as indicated by higher Alpha 2 power at Fz and Mu 2 power), reduced verbal-analytical engagement (higher T7 Alpha 2 power), reduced conscious perception of sensations (higher SMR power), reduced visuospatial processes (higher Alpha 2 power at Pz and T8), and enhanced cognitive control of sustained attention (higher Fz Theta power).

**Discussion:**

These findings support the notion that the achievement of psychomotor efficiency involves the selective activation and inhibition of neuromotor processes. The study outcomes not only contribute to a broader understanding of the refinement of neuromotor processes during the transition from novice to amateur, but also specify neuromotor processes that can be categorized within the framework of psychomotor efficiency.

## 1 Introduction

An examination of the neural activity underlying superior motor performance can provide critical information for specifying the superior neuromotor processes that could facilitate skill acquisition and enhance motor performance. The definition of superior motor performance is an ability to execute motor tasks with exceptional skill, precision, speed, and efficiency compared with others. For example, high-level performance as motor performance in highly-skilled athletes compared with novices appears a smooth and effortless movement (Lay et al., 2002) and an effective way to engage neuromotor processes (Wang et al., 2020). To explain high-level performance, the psychomotor efficiency hypothesis posits that superior motor performance involves a refined set of inputs to the orchestration of central neuromotor processes in the brain (Hatfield, 2018). Specifically, greater motor skill may be characterized by the suppression of task-irrelevant neuromotor processes (e.g., reduced neuromotor noise) and the promotion of essential neuromotor processes to organize the intended action. Akin to previous studies, this hypothesis mirrors the expert–novice and expert–amateur paradigms in a variety of motor tasks, such as golf putting, shooting, and dart throwing (Cheng et al., 2015a; Doppelmayr et al., 2008; Wang et al., 2020). Drawn from the expert–novice paradigm, previous studies have shown, for example, that experts exhibit a global decrease in neural activity in the brain (Cheng et al., 2015a; Filho et al., 2021; Hatfield, 2018) as the suppression of task-irrelevant neuromotor processes during motor preparation. In contrast, Wang et al. (2020) adopted the expert–amateur paradigm to test psychomotor efficiency hypothesis and observed that expert golfers exhibit increase neural activity in specific cortical regions, including premotor cortex that is associated with motor programming and parietal cortex and right-temporal cortex that are associated with visuospatial attention. Based on these findings, Wang et al. (2020) suggested neural activity during superior motor performance are more complex than first thought because the psychomotor efficiency may involve a selective increase in task-relevant neuromotor processes. Although previous studies have separately adopted the expert–novice and expert–amateur paradigms (Cheng et al., 2015a; Filho et al., 2021; Hatfield, 2018; Wang et al., 2020) to specify the achievement of psychomotor efficiency, there is a research problem that remains a lack of detailed understanding of the specific mechanisms through which neural activities contribute to performance efficiency in the early stages of motor skill learning, such as amateur versus novice.

Building upon the findings of expert–novice and expert–amateur comparative studies, further inquiry using a novice–amateur paradigm could shed light on essential neural activities in the early stages of motor skill learning to address this research problem. Specifically, this approach is crucial for gaining a comprehensive understanding of neuromotor processes in motor learning and performance, particularly during the cognitive and associative stages (Fitts and Posner, 1967). From a cognitive perspective (Fitts and Posner, 1967), motor skilled learning can be categorized into three stages: cognitive, associative, and autonomous stages. Accordingly, novice athletes, for example, tend to concentrate on understanding the rules of golf during the cognitive stage of learning (Moran and Toner, 2017). They may engage in extensive thinking and be uncertain about which information is relevant when attempting to execute a movement. As novice golfers progress to the associative stage, they may have already acquired a certain level of proficiency in golf by translating declarative knowledge into procedural knowledge (i.e., from “what to do” to “how to do it”; Anderson, 1982). In this stage, they may shift their attention and cognitive processes toward reducing unnecessary neuromotor processes. Once golfers are in autonomous stage, they are characterized by allocating more attention and refining the necessary neuromotor processes to achieve superior performance (Hatfield, 2018).

The notion was specified using neuroimaging (fMRI) and electroencephalography (EEG). The assessment of brain and neural activities have revealed a dynamic refinement of neuromotor processes mechanism at the different level of skills. For example, Chang et al. (2018) who utilized fMRI and observed that novices had lower resting-state functional connectivity seeded from the right middle temporal pole than amateurs and experts. Furthermore, Chen et al. (2022a) and Chen et al. (2022b) who adopted EEG coherence analysis and observed that novice golfers had higher connectivity in motor-sensorimotor circuit than amateur and elite golfers during motor preparation. Interestingly, amateur golfers exhibited lower circuit in motor-sensorimotor connectivity than elite golfers. Although these findings have specified a nonlinear refinement of functional connectivity in the brain from novice to expert, reflecting increasing specialization and efficiency in task-relevant neuromotor processes, the analyses that they used are limited regarding the assessment of the level of neural activity within a brain region which reflects neuronal excitability.

To understand the refinement of neural activities in preparation for intended action, adopted EEG power analysis is a suitable method for assessing neural excitability, offering valuable insights into various neuromotor processes (Wang et al., 2020). For example, Fz Theta (4–7 Hz at the frontal cortex) is related to the mental effort that requires to sustain their attention during motor task (Doppelmayr et al., 2008). In golf study, Chen et al. (2022a) and Chen et al. (2022b) observed that higher Fz Theta power is associated with an increase in cognitive control of sustained attention, whereas lower Fz theta activity is associated with weaker cognitive control of sustained attention. Similarly, Baumeister et al. (2008) and Haufler et al. (2000) suggested that Pz and T8 Alpha 2 (10–12 Hz at the parietal and the right temporal cortices) are associated with visual–spatial attention. They observed that higher Pz and T8 Alpha 2 power reflect reduced visual–spatial processing during motor preparation (Haufler et al., 2000; Wang et al., 2021). In addition to attention-related neuromotor processes, researchers demonstrated that T7 Alpha 2 (10–12 Hz at the left temporal cortex) have been associated with verbal-analytic processing (Bellomo et al., 2020; Wang et al., 2022; Wang et al., 2023b). Higher T7 Alpha 2 power is associated with reduced verbal-analytic processing, such as consciously planning putting mechanics or providing self-instructional cues for movement (Haufler et al., 2000). In addition, Cheng et al. (2015a) and Wu et al. (2023) investigated the relationship between somatosensory processing and motor performance. They observed SMR (12–15 Hz at the central cortex) reflects somatosensory processing, including the sensation of movement or body position during motor preparation. Other scholars confirmed Fz Alpha 2 (10–12 Hz at the frontal cortex) and Mu 2 (10–12 Hz at the central cortex) have been associated with motor programming processing, such as motor planning and motor control (Babiloni et al., 2008; Cooke et al., 2014; Wang et al., 2019; Wang et al., 2020; Wang et al., 2022; Wang et al., 2023b).

Notably, Mu 2 and Alpha 2 bands reflect different neurocognitive processes. Mu 2 is an EEG rhythm emerging over the sensorimotor regions (Cz) and is distinguishable from the parietal and occipital Alpha 2. Mu 2 power reflects the allocation of cognitive resources in motor programming, especially in motor control (Pineda, 2005) during goal-directed actions and observational tasks (Cannon et al., 2014). In contrast, Alpha 2 power in the parietal and occipital regions has linked to attentional processes (Loze et al., 2001). Although the Mu 2 overlaps with SMR at the same region, they have been associated with different functions (Wang et al., 2019). For example, previous studies have indicated that lower Mu power at Cz was associated with a corrective action relating to previous movement errors (Cooke et al., 2015) and a resulting successful putting performance, particularly during difficult tasks (Babiloni et al., 2008; Cooke et al., 2014; Wang et al., 2019). In contrast, higher SMR power was associated with less sensory input that interference in motor processing (Cheng et al., 2015a; Cheng et al., 2017). That is, a relaxed yet focused state (Wu et al., 2023) and the efficiency of cortical processing during skilled motor preparation in successful putting performance (Cheng et al., 2023; Wang et al., 2019).

Recent evidence demonstrated that neuromotor refinement can be accelerated by modulating these EEG rhythms through neurofeedback training (NFT) interventions. For instance, researchers adopting NFT enhance motor control and attentional focus by modulating SMR (12–15 Hz), alpha (8–12 Hz), and mu (8–13 Hz) wave activity during precision tasks (Cheng et al., 2015a; Chen et al., 2022a; Chen et al., 2022b; Pourbehbahani et al., 2023), resulting in the positive development of psychomotor efficiency (Tosti et al., 2024). A recent systematic review and meta-analysis by Yu et al. (2025) further affirmed the positive impact of NFT on these EEG rhythms across a range of complex sports, revealing consistent improvements in motor accuracy and neural adaptation across training protocols. Given the evidence, researchers emphasize that it is essential to uncover the functional roles of EEG components associated with developing expertise, guiding the creation of EEG targeted interventions to boost skill acquisition.

An increasing amount of evidence has incorporated these components in sports studies. Previous expert–novice studies have demonstrated that experts have higher Fz theta, Fz, Pz, T7, T8 Alpha 2, Mu 2, and SMR power (Baumeister et al., 2008; Cheng et al., 2015a; Cooke et al., 2014; Del Percio et al., 2009; Doppelmayr et al., 2008; Haufler et al., 2000) than novices. These authors suggested that experts appear to exhibit greater cognitive control of sustained attention while relying less on visual–spatial and verbal-analytic processing. Additionally, they allocate fewer cognitive resources to motor programming and sensory input. Interestingly, Wang et al. (2020) adopted the expert–amateur paradigm and observed that the characteristic of elite golfers was with lower Alpha 2 power at Fz, Pz, and T8 as well as lower Mu 2 power, suggesting that elite athletes refine specific neuromotor processes, contributing to superior motor performance. Previous EEG studies only used either the expert–novice paradigm or the expert–amateur paradigm to account for the refinement of neuromotor processes. However, it is important to consider using the novice–amateur paradigm, as it focuses on the critical transition between early-stage learners, providing valuable insights into the gradual refinement of neuromotor processes. Accordingly, using an amateur–novice design that narrows the gap in skill level (for example, at the cognitive and associative stages) can complement existing findings on previous novice–expert (i.e., the cognitive and autonomous stages) and amateur–expert (i.e., the associative and autonomous stages) EEG studies. By doing so, we can further specify critical information on the refinement of neuromotor processes from novices to amateurs (i.e., the cognitive and associative stages) that can enhance the understanding of the achievement of psychomotor efficiency.

Accordingly, we aim to investigate the neural activities underlying the early stages of motor skill learning by measuring EEG power across different frequency bands (Theta, Alpha 2, Mu 2, and SMR) in the golf putting task. To ensure accurate EEG data collection, a golf putting task was used that minimizes muscle artifacts during the motor preparatory period, thus reducing potential interference in the EEG readings (Cooke et al., 2014; Wang et al., 2019). Participants were categorized into two skill groups, following the definition provided by Chen et al. (2022a) and Chen et al. (2022b): amateurs, defined as individuals competing at an intermediate skill level, and novices, who had no prior experience playing golf. By examining the differences in EEG power between these two groups, we seek to provide detailed insights into the specific neural mechanisms involved in the acquisition and refinement of motor skills. Based on the psychomotor efficiency hypothesis (Hatfield, 2018) and previous research findings in the expert–novice paradigm (Baumeister et al., 2008; Callan and Naito, 2014; Cheng et al., 2015a; Del Percio et al., 2009; Doppelmayr et al., 2008; Haufler et al., 2000), our research objective is to determine whether Fz theta, Fz, Pz, T3, T4 Alpha 2, Mu 2, and SMR power can be also used to differentiate between amateurs and novices. As previous studies have shown higher Fz theta, Fz, Pz, T3, T4 Alpha 2, Mu 2, and SMR power in experts compared to novices (Baumeister et al., 2008; Cheng et al., 2015b; Cooke et al., 2014; Del Percio et al., 2009; Doppelmayr et al., 2008; Haufler et al., 2000), we hypothesized that amateur golfers would show higher Fz theta, Fz, Pz, T3, T4, Mu 2, and SMR power compared to novices before action.

## 2 Materials and methods

### 2.1 Participants’ recruitment

Power analysis for the repeated measures multivariate analysis of variance (MANOVA) was conducted using G*Power to calculate the required sample size (Faul et al., 2007). Based on previous studies with similar research design (Chen et al., 2022a; Chen et al., 2022b), the following parameters were used: *α* = 0.05, power = 0.80, effect size = 0.28 (corresponding to η_p_^2^ = 0.33), number of groups (amateur and novice) = 2, and number of measurements (Fz theta, Fz, Pz, T7, and T8 alpha 2, Mu 2, and SMR) = 7. In the approximation method, Wilks’ Lambda (Rao, 1951) and the algorithm by O’Brien and Shieh (1999) were used to compute both the effect size and the sample size, resulting in a minimum sample size of *N* = 16. We recruited 16 amateur golfers from golf clubs (9 females, 7 males, mean age = 20.81 ± 1.83) and 16 novices (9 females, 7 males, mean age = 22.25 ± 1.61) to minimize potential biases in power analysis, a concern that has been highlighted in the neuroscience literature (Albers and Lakens, 2018; Algermissen and Mehler, 2018). The distinction between amateurs and novices in this study was based on multiple criteria, including handicap and years of experience. While amateurs had an average handicap of 33 ± 5.68, novices had no experience with golf, either recreationally or competitively. Furthermore, the amateurs’ mean of 3.81 ± 1.83 years of golf experience supports their classification as intermediate-level golfers. Handicap is widely recognized for distinguishing skill levels. According to United States Golf Association (USGA) statistics, a handicap range of 30–34.9 reflects golfers whose performance is below 96.82% of male and 56.84% of female players nationally (United States Golf Association, 2024). While this range overlaps with the lower end of intermediate players, it reflects a significant gap in skill and experience compared to novices. This categorization aligns with prior studies that define amateur athletes as those who compete and train regularly but do not reach expert-level performance (Scharfen and Memmert, 2019; Swann et al., 2015). To ensure consistency across participants, the inclusion and exclusion criteria were clearly defined and applied separately. Inclusion criteria required participants to be right-handed (assessed via the Edinburgh Handedness Inventory; Oldfield, 1971), aged between 18 and 25 years, possess normal or corrected-to-normal vision, and exhibit normal visual selective attention as measured by the Trail Making–A Test (Partington and Leiter, 1949). In addition, amateurs were required to have a golf handicap between 30 and 36 and at least 2 years of regular practice, whereas novices had no prior golf experience. Exclusion criteria included any history of neurological or psychiatric disorders, current use of medications affecting the central nervous system, and consumption of alcohol or caffeine within 24 h prior to the experimental session. This study was approved by the institutional review board of Bielefeld University. All of the procedures were carried out according to the relevant guidelines and regulations of the Research Ethics of the 6^th^ Edition of the Helsinki Declaration.

### 2.2 Study measures

#### 2.2.1 Golf putting task

**Figure 1:**
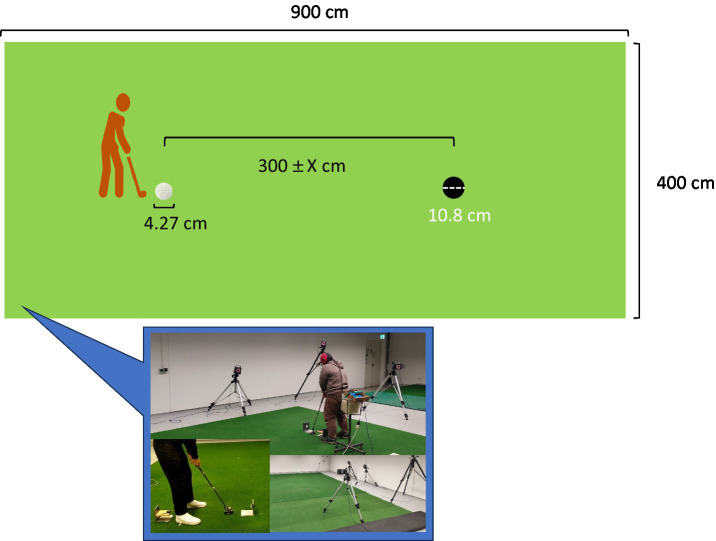
Schematic diagram of the laboratory. “X” is based on the putting success rate in the warm-up trial. More detail please refer to Figure 2.

**Figure 2:**
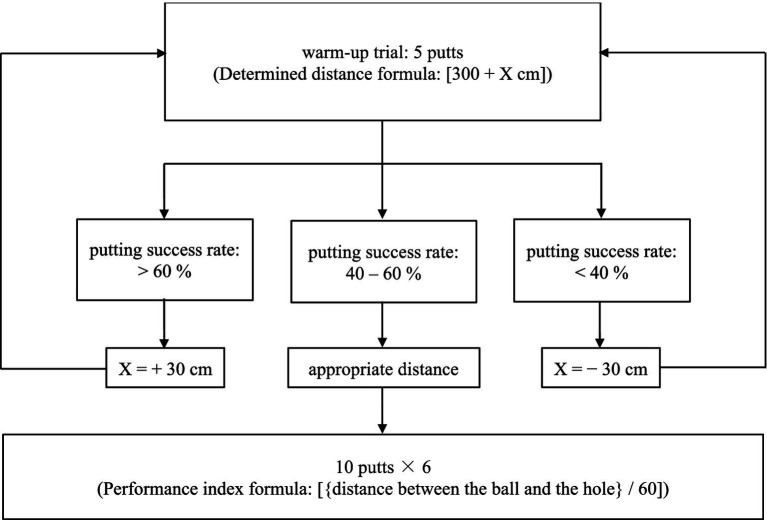
The process of golf putting task.

Participants were asked to perform the putting task that was executed in the laboratory on an artificial putting green that consisted of a green (900 × 400 cm), and a standard-sized golf hole (diameter = 10.8 cm). The distance between the starting point of the ball (4.27 cm diameter) and the hole was determined 40–60% of all putts, which would be missed for each of the participants during warm-up trials (Chen et al., 2022a; Chen et al., 2022b; Wang et al., 2020). Specifically, all participants putted 300 cm in the beginning distance. Next, participants performed 5 putts, and the distance was adjusted relying on whether the average of 5 putting success rate was within 40–60%. If the success rate was fell between 40 and 60%, the putting distance was set at 300 cm. If the success rate was above 60% or below 40%, the putting distance was increased 30 cm or decrease 30 cm. Afterwards, participants were asked to performed extra five putts to ensure that the success rate reached 40–60%. After the appropriate putting distance was decided, the participants started to perform 60 putts with the average distance related to 40–60% success. The definition of the motor preparation period was consistent with that specified by Wang et al. (2019) who defined it as the time between placing the putter behind the ball and initiating the backswing. Event marker data were initiated via an infrared sensor that detected the movement of the backswing (Figures 1, 2).

#### 2.2.2 Subjective anxiety level

To prevent confounding effects of anxiety, the individuals were asked to report a feeling of anxiety level with a visual analogue scale (VAS; Wang et al., 2020). In the VAS of anxiety level, a scale-line from “no anxiety at all” (0 score) to “highest anxiety level” (10 score) was set during each rest period throughout the golf putting task.

### 2.3 Vicon motion systems

In this study, the recording of putting performance was conducted utilizing a motion capture system for determining individual golf putting distance, namely Vicon Motion System (Oxford, UK). The system consisted of six T10 charge-coupled device cameras, which were employed to track the movement of the ball during rolling and stopping phases. The Vicon system provides a spatial resolution of approximately 0.25 mm and a temporal resolution of 200 Hz, ensuring precise tracking during the task. Once the ball stops, the system calculates the distance between the ball and the hole, allowing for detailed analysis of performance (Wang et al., 2023a).

### 2.4 EEG recording

**Figure 3:**
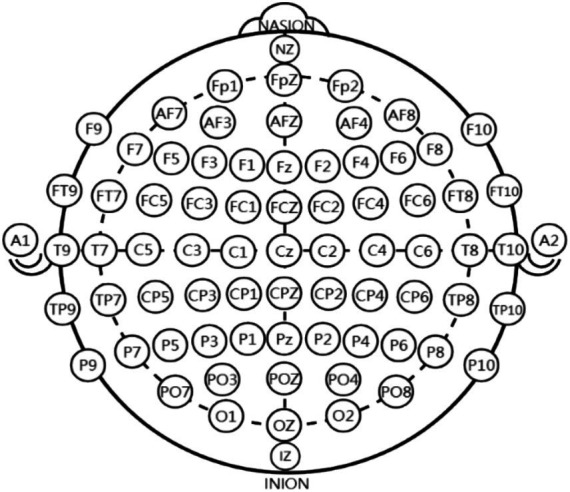
Electrode placement according to the international 10–10 EEG system, illustrating the standardized distribution of scalp electrodes used for recording cortical activity.

To record the EEG activity, an electro cap was used to record and followed the international 10–10 system, with 64 electrode sites recorded in total (Figure 3). The electrical reference was located on the left and right ear mastoids (M1, M2), and the ground electrode was located at the anterior frontal zone position (AFz; Jurcak et al., 2007). The vertical and horizontal electrooculograms (HEOL, HEOR, VEOU, and VEOL) were recorded with bipolar configurations located superior and inferior to the left eye and on the left and right orbital canthi. The eego system (ANT Neuro, Germany) was used with a bandpass filter from 1 to 100 Hz and a 50 Hz Notch filter. The eego software was used to collect data with a sampling frequency of 500 Hz. Electrode impedance was kept below 10 kΩ.

### 2.5 Experimental procedure

**Figure 4:**
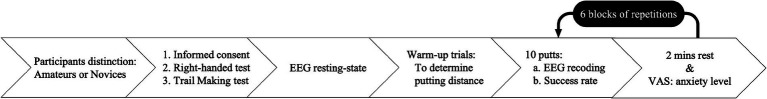
Experimental process schematic diagram.

The experimental procedure followed the protocol established by Wang et al. (2020) to assess participants’ performance and EEG activity. All participants were asked to abstain from alcohol and caffeine for 24 h before the testing day. On the testing day, they were first explained the nature of the study and asked to sign an informed consent form. After agreeing to participate, they were asked to complete the right-handed and Trail Making–A Tests. Next, they wore a Lycra electrode cap and kept their eyes open to gaze at the ball for 90 s in preparation for recording resting-state EEG. Following this, they completed warm-up trials to determine their individual putting distance, starting at 300 cm. They performed 5 trial putts, and if their success rate was outside the hole range of 40–60%, the distance was adjusted by 30 cm, either up or down. This process continued until the hole success rate was achieved (Wang et al., 2022; Wang et al., 2023b). Once the appropriate putting distance was established, participants performed 10 putts per block across 6 blocks, with a 2-min rest between each block. The entire session lasted approximately 90 min to minimize fatigue effects on EEG readings (Wang et al., 2019). During rest periods, participants reported their subjective anxiety levels using a VAS. The procedure is illustrated in Figure 4.

### 2.6 EEG data management

#### 2.6.1 Behavioral data

In order to assess the performance outcomes, the calculation of putting accuracy was conducted through the utilization of the mean radial error (MRE) in putting performance analysis, as proposed by Wang et al. (2022) and Wang et al. (2023b). The MRE is defined as the mean distance (mm) between the putt outcomes of each participant and the center of the designated golf hole.

#### 2.6.2 EEG data

The EEG data were preprocessed by using EEGLAB functions (Delorme and Makeig, 2004) and using custom scripts written in MATLAB (MathWorks, U. S. A.). The EEG preprocessing steps consisted of (1) re-referencing the EEG data to the averaged mastoids (M1, M2); (2) setting the bandpass filter from 1 Hz (low-pass) to 30 Hz (high-pass) using a basic finite infinite response (FIR) filter; (3) extracting epochs from the − 3,000 to 1,000 ms time window before putting; (4) removing channels with bad signals; (5) rejecting gross artifacts (amplitudes exceeding ± 100 μV) to eliminate any potential biological artifacts (e.g., muscle activation artifacts; Wang et al., 2020); (6) running independent component analysis (ICA; Runica Infomax algorithm; Makeig et al., 1996) to identify and remove components arising from blinks, eye movements, and other non-neural activity; (7) interpolating channels with bad signals; (8) dividing the clean signals into 2-s epochs (− 2,000 to 0 ms before putting); and (9) The power spectrum was calculated by using Welch estimation method (Hanning windowing function; Welch, 1967). Considering potential individual differences in brain activity that could potentially confound our results, we assessed Individual Alpha Peak Frequency (IAPF) during resting state in each participant. IAPF is defined as the maximum power value in the EEG frequency spectrum between 7.5 and 12.5 Hz (Klimesch, 1999). For healthy adults IAPF lies between 9.5 and 11.5 Hz (Klimesch, 1999). Accordingly, the selected frequency bands were as follows: theta (IAF –6 to IAF –3 Hz), alpha 2 and Mu 2 (IAPF to IAPF+2 Hz), as well as SMR (IAPF +3 to IAPF+5 Hz). The mean IAPs were 9.90 ± 0.65 Hz and 9.94 ± 0.66 Hz for the amateurs and novices, respectively. An independent *t*-test showed no significant difference in the mean IAPFs between the two groups (*p* = 0.837).

### 2.7 Statistical analysis

A total of four statistical analyses were conducted.

#### 2.7.1 Putting performance

Two separate one-way ANOVAs were conducted, one on the distance of the golf ball from the hole and the other on the success rate of golf putts.

#### 2.7.2 EEG power

Based on previous EEG studies, a one-way MANOVA was conducted with 2 groups (amateur and novice) as the independent variable on Fz theta, Fz, Pz, T7, and T8 alpha 2, Mu 2, and SMR as the dependent variables.

#### 2.7.3 Comparing correlation coefficients

To examine whether the strength of the MRE–EEG relationships becomes stronger as motor skill level increases, Fisher’s *r*-to-*z* transformations were conducted to compare the correlation coefficients between groups (Howell, 2009) across eight components: Fz theta, Fz alpha 2, Mu 2, Pz alpha 2, T7 alpha 2, T8 alpha 2, and Cz SMR. Following the transformation, z scores from each group were contrasted using a standard z-test for independent correlations. The standard error of the difference was calculated based on sample sizes, following the method proposed by Fisher (1921). The resulting z statistics were then converted into *p* values to assess the statistical significance of group differences.

#### 2.7.4 Control analysis

##### 2.7.4.1 VAS anxiety level

To ensure whether anxiety levels may be a potential confounding, VAS anxiety level was compared between and within participants during the golf putting task using a two-way ANOVA.

##### 2.7.4.2 Task specificity

To determine whether the EEG power was task-specific in the golf putting task, the EEG power in the resting condition was analyzed. The continuous EEG data were segmented into 2-s epochs to obtain the mean EEG power in the resting condition. Statistical analyses were conducted for the EEG measures Fz theta, Fz, Pz, T7, and T8 alpha 2, Mu 2, and SMR using a two-way MANOVA.

When the multivariate effect was significant, univariate ANOVAs were used to examine the differences between the groups in each measure. Analyses with between-subjects levels used the Wilks’ lambda statistic. Furthermore, effect size estimates were calculated from the partial *η*^2^. In case of significance in the post-hoc analysis, false discovery rate (FDR) was used to control potential inflation of the Type I error value due to the multiple comparisons. The alpha level was set at 0.05 for all analyses before FDR (Genovese et al., 2002).

## 3 Results

### 3.1 Behavior results

A one-way ANOVA analysis with distance of the golf ball from the hole showed that the groups differed in the putting distance, *F*(1, 30) = 58.78, *p* < 0.001, with the amateurs’ putting distance (M = 351 ± 28 cm) being longer than novices’ (M = 248 ± 45 cm). However, there was no significant difference in the success rate of golf putts between two groups, *F*(1, 30) = 0.641, *p* = 0.430. That is, we successfully controlled the task difficulty in both groups.

### 3.2 EEG power

**Figure 5:**
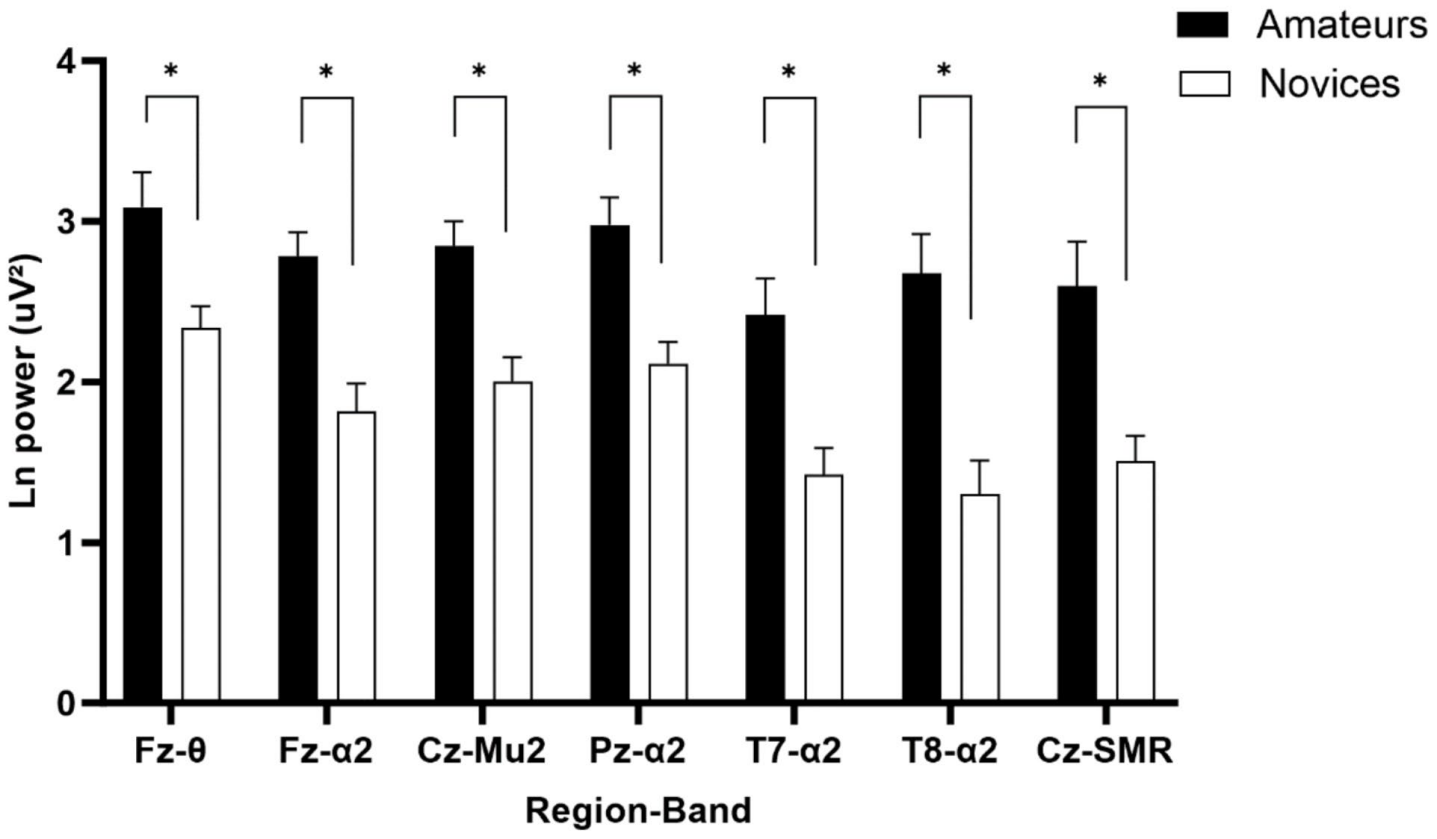
Mean values for theta (4–7 Hz), alpha 2 (10–12 Hz), mu2 (10–12 Hz), and SMR (12–15 Hz) power in the amateur and novice groups for Fz, Cz, Pz, T7, and T8. Error bars represent standard errors. *Significant difference, *p* < 0.05 (FDR corrected).

A one-way MANOVA analysis with a 2 (Group: amateur, novice) as independent variable on Fz theta, Fz, Pz, T7, and T8 alpha 2, Mu 2 and SMR as dependent variable yielded a significant group effect, *F*(7, 24) = 2.875, *p* = 0.025, *λ* = 0.544. *η_p_^2^* = 0.456. As can be seen in Figure 5, the univariate ANOVAs showed that the groups differed statistically on Fz theta, *F*(1, 30) = 8.562, *p* = 0.006, *η_p_^2^* = 0.222; Fz alpha 2, *F*(1, 30) = 17.776, *p* < 0.001, *η_p_^2^* = 0.372; Mu 2, *F*(1, 30) = 15.496, *p* < 0.001, *η_p_^2^* = 0.341; Pz alpha 2, *F*(1, 30) = 15.903, *p* < 0.001, *η_p_^2^* = 0.346; T7 alpha 2, *F*(1, 30) = 12.433, *p* = 0.001, *η_p_^2^* = 0.293; T8 alpha 2, *F*(1, 30) = 18.238, *p* < 0.001, *η_p_^2^* = 0.378; and Cz SMR, *F*(1, 30) = 11.729, *p* = 0.002, *η_p_^2^* = 0.281. These results are consistent in showing that all power in amateurs were higher than the novices.

### 3.3 Correlation coefficients

As can be seen in Table 1, among the eight EEG components examined, the correlation at Pz alpha 2 between the amateur group (*r* = 0.374) and the novice group (*r* = −0.297) reached statistical significance based on the uncorrected *p* value (*z* = −1.783, *p* = 0.037); however, this effect did not remain significant after FDR correction (*p* = 0.259). Other components showed no significant but consistent trends in the amateur group compared with novice group for Fz theta (*p* = 0.082, FDR corrected *p* = 0.287), Fz alpha 2 (*p* = 0.406, FDR corrected *p* = 0.406), Mu 2 (*p* = 0.169, FDR corrected *p* = 0.395), T7 alpha 2 (*p* = 0.082, FDR corrected *p* = 0.287), T8 alpha 2 (*p* = 0.253, FDR corrected *p* = 0.443), and Cz SMR (*p* = 0.196, FDR corrected *p* = 0.392). Although none of the comparisons remained significant after FDR correction, the consistent direction of higher EEG–MRE correlations in the amateur group suggests a potential trend toward stronger and more stable brain–behavior coupling as motor skill level increases.

**Table 1:** Group comparison of correlations between EEG power and MRE (Fisher’s *r*-to-*z* test).

EEG component	*r*_NG_	*r*_AG_	*z*	*p*
Fz theta	−0.032	0.473	−1.392	0.287
Fz alpha	0.039	0.132	−0.239	0.406
Cz alpha	−0.046	0.319	−0.960	0.395
Pz alpha	−0.297	0.374	−1.783	0.259
T7 alpha	−0.061	0.451	−1.395	0.287
T8 alpha	−0.015	0.241	−0.665	0.443
SMR Cz	−0.073	0.257	−0.857	0.392

*r* = Pearson correlation coefficient between EEG power and MRE. NG, novice group; AG, amateur group. z = Fisher’s *r*-to-*z* comparison between groups. *p* < 0.05 (FDR corrected).

### 3.4 Control analyses

#### 3.4.1 VAS anxiety level

The VAS-anxiety level was compared between and within subjects during the golf putting task. A two-way ANOVA mixed design 2 (Group: amateurs, novices) × 6 (Block: 1, 2, 3, 4, 5, 6) showed no significant interaction effect between Group and Block, *F*(5, 150) = 0.661, *p* = 0.585, *η_p_^2^* = 0.022 nor a main effect of the Block factor (*p* = 0.074).

#### 3.4.2 Task specificity

A one-way MANOVA was analyzed the EEG measures of Fz theta, Fz, Cz, Pz, T3, T4 alpha 2, and SMR in the resting condition. The result indicated no significant group effect in resting EEG state, *F*(7, 24) = 1.157, *p* = 0.363, λ = 0.748. *η_p_^2^* = 0.252. Therefore, our main finding in EEG was task specific in golf putting between amateurs and novices.

## 4 Discussion

The objective of this study was to determine whether Fz theta, Fz, Pz, T3, T4 Alpha 2, Mu 2, and SMR power can be also used to differentiate between amateurs and novices. The present study compared amateur and novice golfers on EEG 4–7 Hz (Theta), Alpha 2, Mu 2 (10–12 Hz), and SMR (12–15 Hz) power. The main findings of this study were that amateur golfers, compared with novices, were characterized by higher Fz Theta (frontal cortex), Fz, Pz, T7, T8 Alpha2 (frontal, parietal, left temporal, and right temporal cortices), Mu 2 at Cz (central cortex), and SMR at Cz power before the intended action. Current findings complemented the findings of expert–novice and expert–amateur studies by further specifying essential information on the refinement of neuromotor processes between novice and amateurs to better understanding of the achievement of psychomotor efficiency in the early stages of motor skill learning.

The findings are generally in line with the principles of the psychomotor efficiency hypothesis, which postulates that a refinement of brain processes may be associated with two principles: (1) selective inhibition of task-irrelevant neuromotor processes and (2) selective functional activation of neuromotor processes (Hatfield, 2018). Specifically, we observed that amateurs compared with novices had higher Fz Alpha 2 and Mu power reflecting decrease in motor programming, such as motor planning and motor control during motor preparation (Babiloni et al., 2008; Cooke et al., 2014; Li et al., 2025; Wang et al., 2019, 2020). Similarly, amateurs also had higher T7 Alpha 2 reflecting less verbal-analytical engagement before action (Haufler et al., 2000). These findings support the first principle of the psychomotor efficiency hypothesis and are consistent with previous expert-beginner studies. For example, Cooke et al. (2014) observed that experts, compared with beginners, exhibited higher Alpha 2 power at Fz and Mu 2 during the early stages of movement preparation. Cooke and co-workers suggested that experts require fewer cortical resources to organize and control movement during the execution of goal-directed actions (Pfurtscheller, 1992, 2003) due to their expertise in specific tasks (Cooke et al., 2014, 2015). In comparing individuals with expertise in marksmanship to novices, Haufler et al. (2000) found that highly skilled marksmen exhibited higher T7 Alpha 2 power, indicating reduced engagement in verbal-analytical processes (such as internal self-talk) for having a good quality of attention during motor control, when compared with novices. Accordingly, current finding extends previous research on expert-beginner and expert-novice comparisons by demonstrating that reducing motor programming (higher Fz Alpha 2 and Mu 2 power) and verbal-analytical engagement (higher T7 Alpha 2 power) may be also crucial for transitioning from being a novice to an amateur.

Further, amateurs had higher SMR power than novices. SMR is considered as sensory processing which refers to the sensory input during motor preparation, including one’s own body and the environment. SMR shows a negative correlation with somatosensory and motor cortical activities (Mann et al., 1996). Increase in SMR power has been associated with inhibition of sensory information that may decrease the sensory input, facilitating relaxed attention focusing to improved motor performance (Wu et al., 2023). This notion is supported by expert-novice comparison. For example, Cheng et al. (2015a) found experts relatively had higher SMR power than novices before action, suggesting that experts may rely to a lesser extent on somatosensory information processing (e.g., the sense of body position and movement) to execute their throwing movement in a comparatively more adaptable manner. Conversely, novices may tend to utilize feedback derived from kinesthetic information (e.g., the body’s position, movement, and orientation in space) to carry out the throwing task. Thus, the present finding further expands Cheng et al.’s (2015a) study by demonstrating that higher SMR power was still observable when amateur golfers are compared to novices. That is, amateurs may also depend on reduced sensory input in order to prevent any disruption in attentional focus during motor preparation.

Besides motor programming, verbal-analytical, and sensory processes, attentional processes are also critical for the achievement of psychomotor efficiency. The present study found that amateurs, compared with novices, exhibited higher Alpha 2 power at Pz and T8. Alpha 2 power at these regions has been associated with visuospatial processes (i.e., focusing on specific visual and spatial information in the environment) (Balslev et al., 2005; Corbetta et al., 2000; Romei et al., 2010; Wang et al., 2021). Higher Alpha 2 power at these regions reflects the need for fewer neuronal resources in visuospatial processes during a task. In golf, Baumeister et al. (2008) observed that experts, compared with novices, exhibited higher Alpha 2 power at Pz, but not at T8, during a golf putting task. This suggests that novices may need to actively process unfamiliar cues, requiring more neuronal resources at Pz. Interestingly, Wang et al. (2020) found that amateur golfers, compared with elite golfers, have higher Alpha 2 power at Pz and T8, suggesting elite golfers have refined specific visuospatial processes at these regions. Our findings further extend previous studies (Baumeister et al., 2008; Wang et al., 2020) by comparing amateurs with novice. We suggest that higher Alpha 2 power in these regions may be associated with the selective inhibition of task-irrelevant neuromotor processes, thereby contributing to improved performance efficiency in the early stages of motor skill learning.

We also found that amateurs exhibited higher Theta (4–7 Hz) at Fz, which extends the previous literature on the amateur–novice paradigm. For example, in sports, Haufler et al. (2000) and Doppelmayr et al. (2008) found higher Fz theta power in marksmen compared with novice during a shooting task. Fz Theta has been associated with top-down processing of sustained attention (i.e., the ability to maintain focus and remain attentive to a task; Eschmann et al., 2018) and is positively related to mental effort, which is necessary for tasks requiring sustained attention (Chen et al., 2022a; Chen et al., 2022b; Yu et al., 2024). Higher Fz Theta power reflects an increase in cognitive control of sustained attention, suggesting that individual maintain focused attention over a prolonged period through enhanced mental effort. In contrast, lower Fz theta power indicates reduced cognitive control of sustained attention (Cavanagh and Frank, 2014; Sauseng et al., 2007). Accordingly, compared with novices, we suggest that amateurs are characterized by stronger cognitive control of sustained attention during a golf putting task, as they selectively activate task-relevant processes. These findings support the second principle of the psychomotor efficiency hypothesis.

Taken together, the above findings reveal a clear picture of the neuromotor processes in superior performance, especially during the cognitive and associative stages of learning, as seen in novices and amateurs (Fitts and Posner, 1967). Before putting, amateurs were characterized by reduced motor programming (higher Alpha 2 power at Fz and Mu 2 power), reduced verbal-analytical engagement (higher Alpha 2 power at T7), reduced conscious sensation of movement (higher SMR power), and reduced visuospatial processing (higher Alpha 2 power at Pz and T8). Additionally, amateurs demonstrated enhanced cognitive control of sustained attention (higher Theta power at Fz). Beyond these spectral features, the amateur group showed stronger EEG–MRE correlations compared with the novice group. Although none of the components remained significant after FDR adjustment, the consistent direction of effects suggests a potential trend toward stronger and more stable brain–behavior coupling as motor skill level increases (Dayan and Cohen, 2011). This pattern may reflect the emergence of a more reliable association between cortical activity and motor output during skill acquisition. These findings not only support the two principles of neuromotor efficiency hypothesis, but also specified the neuromotor processes underlying superior performance in the early stages of motor skill learning. Importantly, our findings complemented previous literature on expert–novice (i.e., the autonomous and the cognitive stages) and expert–amateur (i.e., the autonomous and the associative stages) paradigms. We suggested that the refinement of neuromotor processes as the achievement of psychomotor efficiency may be associated with a potential inverted U shape neural activity according to the stage of learning (Chang et al., 2018; Chen et al., 2022a; Chen et al., 2022b; Wang et al., 2020).

### 4.1 Control analyses

The control analyses supported our findings on the EEG parameters, which differentiated amateurs from novices during the golf putting condition, were task-specific, as no group differences in Fz Theta, Fz, Pz, T7, T8 Alpha 2, Mu 2, and SMR power were observed in the resting condition. We suggest that these two groups adopted different neurocognitive strategies (e.g., attentional and motor programming processes) possibly as an adaptation to the demands of the task through long-term practice.

## 5 Limitations

This study has several potential limitations that should be considered. First, the study focused specifically on golfers as a complex motor skill, and it is unclear whether the observed findings would generalize to other types of motor skill, such as simple motor task (i.e., air pistol shooting; Wang et al., 2022; Wang et al., 2023b). Future research should explore the applicability of these findings to different motor skill domains. Second, the present study is a cross sectional research, which limits to draw causal inferences. To address this limitation, longitudinal studies are needed, as demonstrated in Wang et al. (2024), to examine the developmental trajectory of neuromotor processes using EEG from novices to amateurs over time. Third, it is essential to interpret the particular neuromotor mechanisms that are associated with selective neurophysiological activities because the “known” psychological event was not manipulated directly in our study. Future research should adopt an approach that compares the well-known psychological state of different mental states, such as focus of attention manipulations (i.e., external focus and internal focus; Wang et al., 2022; Wang et al., 2023b). By doing so, the specific neuromotor processes can be inferred to understand the “unknown” psychological processes. Fourth, no causal relationship between these neuromotor processes and superior performance in the early stages of motor skill learning due to the cross-sectional design of this study. Manipulation of these neuromotor processes through neurofeedback training to examine the effects on motor performance is encouraged for future studies. Lastly, while this study focused on theta, alpha, mu, and SMR bands, other EEG rhythms—such as beta (13–30 Hz) and gamma (>30 Hz)—remain underexplored. Bichsel et al. (2021) observed that beta activity is linked to motor control and has shown promise in improving motor initiation in clinical populations. Similarly, increased gamma power has been associated with reduced feature binding costs and improved intelligence, suggesting its role in cognitive-motor integration (Keizer et al., 2010). Future studies should investigate the potential of beta and gamma that may be associated with superior performance, particularly in athletic settings.

In terms of practical implementation in visuomotor skills learning (e.g., golf putting), it is important to consider emerging neurofeedback interventions that target EEG spectral components as a means of accelerating motor skill acquisition. Previous studies have shown that structured NFT protocols focusing on SMR and Mu enhancement or theta–alpha training can not only modify neural signatures associated with motor preparation but also improve behavioral outcomes such as accuracy and stability (Cheng et al., 2015a; Wang et al., 2022; Wang et al., 2023b; Wu et al., 2023). These results align with the present findings, indicating that amateurs—who may have benefited from prior motor training—exhibit EEG profiles that are also trainable via NFT. Moreover, the evidence from NFT research strengthens the psychomotor efficiency hypothesis by demonstrating that targeted modulation of EEG components can contribute to more efficient neuromotor engagement (Cheng et al., 2024; Tosti et al., 2024). Thus, integrating NFT-based approaches may offer a promising avenue for practical application in early-stage athletes.

## 6 Conclusion

In summary, the findings indicate that, compared with novices, amateurs were characterized by reduced motor programming processes in motor planning and control, reduced verbal-analytical engagement, decreased visuospatial processes, reduced sensation of movement, and enhanced cognitive control of sustained attention. These results provide important insights into the two main tenets of the psychomotor efficiency hypothesis: (1) the selective inhibition of task-irrelevant neuromotor processes and (2) the selective functional activation of neuromotor processes during the transition from novice to amateur. These results not only provide insights into the refinement of neuromotor processes in amateur athletes, but also underscore the potential of neurofeedback training as a practical intervention to accelerate the development of psychomotor efficiency in early-stage learners.

## Data Availability

The raw data supporting the conclusions of this article will be made available by the authors, without undue reservation.
